# Detection of African swine fever virus utilizing the portable MatMaCorp ASF detection system

**DOI:** 10.1111/tbed.14411

**Published:** 2021-12-21

**Authors:** Mariceny Zurita, Lauren Martignette, Jose Barrera, Michael Carrie, Heather Piscatelli, Alyssa Hangman, David Brake, John Neilan, Dustin Petrik, Michael Puckette

**Affiliations:** ^1^ SAIC (formerly with Leidos) Plum Island Animal Disease Center Greenport New York; ^2^ MatMaCorp Lincoln Nebraska; ^3^ BioQuest Associates, LLC Plum Island Animal Disease Center Greenport New York; ^4^ US Department of Homeland Security Science and Technology Directorate Plum Island Animal Disease Center Greenport New York

**Keywords:** African swine fever, amplification, detection, food safety, Solas 8, tissues

## Abstract

African swine fever (ASF) has emerged as a major threat to domestic and wild suid populations, and its continued spread threatens commercial swine production worldwide. The causative agent of ASF, African swine fever virus (ASFV), possesses a linear, double stranded DNA genome. Traditional detection of ASFV relies on laboratory‐based virus isolation or real‐time PCR of samples, typically blood or spleen, obtained from suspect cases. While effective, these methodologies are not easily field deployable, a major limitation during disease outbreak and response management scenarios. In this report, we evaluated the MatMaCorp Solas 8^®^ ASFV detection system, a field deployable DNA extraction and fluorescent detection device, for its ability to extract and detect ASFV from multiple sample types obtained from domestic swine experimentally infected with ASFV strain Georgia. We found that the MatMaCorp Solas 8^®^ ASFV detection device, and affiliated MagicTip™ DNA extraction and C‐SAND™ assay kits, readily detected ASFV in blood and spleen, as well as other sample types, including pinna, liver, skin, muscle and bone marrow.

## 1 INTRODUCTION

African swine fever virus (ASFV) is the only member of the *Asfarviridae* family and causative agent of African swine fever (ASF), an acute haemorrhagic disease with mortality in domestic swine that can approach 100% with highly virulent strains. ASFV has a linear, double stranded DNA genome around 170–190 kb containing at least 150 open‐reading frames.

ASF was first described in Kenya in 1921 (Eustace Montgomery, 1921), and is endemic in sub‐Saharan Africa where it persists in wild suids, notably the African warthog (Anderson et al., 1998; Dixon & Wilkinson, 1988; Heuschele & Coggins, 1965; Luther et al., 2007). There are sporadic, and some long‐term, outbreaks outside of original endemic areas (Bech‐Nielsen et al., 1995; Danzetta et al., 2020; Moura et al., 2010). A 2007 outbreak of ASF in the Republic of Georgia has progressed largely unabated in wild pigs throughout Eurasia and recently into the Caribbean with outbreaks in Dominican Republic and Haiti (Gogin et al., 2013; Gonzales et al., 2021; Maciulskis et al., 2020; Pejsak et al., 2018; Sauter‐Louis et al., 2020; Woonwong et al., 2020; Zhou et al., 2018). The continued spread of ASF has made developing effective countermeasures, such as rapid, field‐deployable detection tools, critical.

The ongoing outbreaks in Eurasia have largely resulted from transportation of contaminated pork products and swill feeding (Flannery et al., 2020; Gogin et al., 2013). Worldwide, movement of pork products from areas containing ASFV represents a major threat for introduction to disease‐free regions (Jurado et al., 2019), and includes international travellers transporting ASFV‐positive pork products (Kim et al., 2019; Wang et al., 2019). ASFV can persist in uncooked pork products for significant amounts of time, survive aspects of meat curing (Botija, 1982; Mebus et al., 1997; McKercher et al., 1978; McKercher et al., 1987), and low infectious doses of ASFV can induce disease in swine (Niederwerder et al., 2019).

While monitoring pork products can help prevent the introduction of ASFV, disease containment requires an expansion of the sample types that may require testing. Reported outbreaks often result in large numbers of dead swine, both feral and domestic, and proper disposal of infected or exposed carcasses is required to prevent further spread. While burial provides time for virus inactivation, (Zani et al., 2020), unburied carcasses still present an opportunity for viral spread, and field carcasses of deceased swine often require on‐site investigations. Carcass conditions, such as the state of decomposition and potential disturbance by scavenger animals, can influence both the quality and availability of samples for testing. Although testing of spleen tissue from swine is the preferred means of detection in terminal cases, more abundant tissues, such as muscle, or protected tissues, such as bone marrow, may be the only available samples in a field setting.

The World Organization for Animal Health (OIE) recommends the use of validated real‐time PCR for the diagnosis of ASF (Aguero et al., 2003; Fernandez‐Pinero et al., 2013; King et al., 2003; OIE, 2019). Unfortunately, real‐time PCR detection methodologies are usually not field deployable. In the present study the MatMaCorp detection system, initially developed for genotyping and endemic animal disease detection, was investigated as a padlock probe‐based assay for ASFV detection. The MatMaCorp ASF detection system comprises the field deployable Solas 8® device and utilizes commercially available MagicTip™ DNA extraction and C‐SAND™ Assay kits, for DNA detection with fluorescent padlock oligonucleotide probes in a hybrid amplification and isothermal assay. The C‐SAND™ assay is a 2‐step process, consisting of padlock probe hybridization and ligation followed by isothermal rolling circle amplification, and optionally preceded by an initial PCR step to enhance detection assay sensitivity.

We evaluated a MatMaCorp ASFV detection system prototype for its ability to extract and detect viral DNA from blood, oral fluid and various tissues harvested from domestic swine experimentally infected with ASFV Georgia (genotype II). The MatMaCorp ASFV detection system is comprised of three components, the MagicTip™ DNA extraction kit, the C‐SAND™ Assay kit, and the Solas 8 device on which both DNA extraction and the detection assays are performed. Components of the MagicTip™ and C‐SAND™ kits can be lyophilized and are stored at room temperature, removing the need for cold storage in field settings. The Solas 8 device is capable of operating on standard AC power, DC power with an inverter, or from a battery pack. Two different C‐SAND™ Assay kit versions, Standard ASFV C‐SAND™ (standard) and High‐Sensitivity ASFV C‐SAND™ (High Sensitivity) were tested. The Standard assay is a 2‐step assay while the High‐Sensitivity assay was modified to include a pre‐amplification step to enhance assay sensitivity. All studies were performed on a Solas 8® device in the laboratory that can be adapted to field use applications.

## 2 MATERIALS AND METHODS

### 2.1 Collection of samples from swine

All ASFV‐positive swine were experimentally infected with ASFV strain Georgia as part of several ongoing studies at the Plum Island Animal Disease Center (PIADC). No swine were infected and euthanized exclusively for this study. No swine received any treatments prior to infection with ASFV. Animals were euthanized upon the appearance of clinical ASF, typically 4–7 days post‐infection. ASFV‐negative tissues were obtained from swine infected with foot‐and‐mouth disease virus as part of a separate ongoing program at PIADC.

#### 2.1.1 Collection of oral fluid with chew ropes

Oral fluid was collected using an oral fluid sample collection kit (IDEXX). Chew ropes were distributed in the pen with infected swine. Oral fluid was collected at 1, 3 and 5 days post‐infection with ASFV and stored at −70°C until testing.

#### 2.1.2 Collection of oral swabs

Sterile cotton tipped applicators (Puritan) were collected by swabbing the inside of the mouth after euthanasia. Swabs were placed in 2.0 ml tubes (Sarstedt) containing media (1× Dulbecco's Modified Eagle Medium, 2% antibiotic‐antimycotic solution, 1% fetal bovine serum) and broken to allow for tube closure. Samples were stored at 4°C until testing.

#### 2.1.3 Blood collection

##### Whole blood

Whole blood samples from infected swine were collected in ethylenediaminetetraacetic acid (EDTA) tubes immediately prior to euthanasia.

##### Blood spots obtained from blood cards

Blood spots were collected on Whatman® FTA® Elute cards (GE Healthcare Life Sciences) as per manufacturer's protocols.

#### 2.1.4 Tissue collection

Tissues were collected from 11 individuals. Due to experimental constraints not all samples were obtained from all individuals. Femoral bone marrow (bone marrow), liver, superficial inguinal lymph node, biceps femoris (muscle), left ear pinna (pinna), abdominal skin (skin), spleen and tonsil were collected from swine that had been humanely euthanized upon meeting the clinical definition of ASF disease. All tissues were collected between 6 and 48 h post‐euthanasia with carcasses stored at 4°C prior to tissue being collected. After tissue collection, samples were aliquoted and stored at 4°C for short‐term storage and at −70°C for long‐term storage.

### 2.2 Incubation of tissues at 37°C to mimic decomposition

To rapidly simulate an advanced state of decomposition, samples of tissues were placed in 50 ml conical tubes and stored at 37°C, 5% CO_2_ for up to 72 h. Within 24 h of incubation, tissues presented a strong pungent odour indicative of a decomposed state.

### 2.3 Extraction using MagMax™ DNA extraction kit

ASFV DNA was extracted using the MagMax™ DNA extraction kit (Thermo), in a 96‐well format as per manufacturer's instructions.

### 2.4 Extraction using MagicTip™ DNA extraction kit

Extraction of DNA using MagicTip™ DNA extraction kits (MatMaCorp), followed manufacturer's instructions. Kits were utilized based on sample type; blood was extracted using the MagicTip™ Blood Isolation kit, oral fluid extracted using the MagicTip™ Oral Fluid Isolation kit, and samples from bone marrow, liver, lymph node, muscle, pinna, skin, spleen and tonsil were extracted using the MagicTip™ Tissue (Animal) Isolation kit. Tissue samples were cut using either a scalpel or sterile punch prior to extraction. Samples were added to a 1.5 ml Eppendorf tube with TL buffer and heated on the Solas 8 device. TN buffer was added and the sample was mixed using the kit's MagicTip glass rod that also served to homogenize tissue samples. The MagicTip, carrying the extracted DNA, was transferred to a second tube with TE buffer and again heated on the Solas 8. Supernatant was removed and tested directly by either traditional Real‐Time PCR or using C‐SAND™ assay kits.

### 2.5 Extraction of DNA from blood cards

Approximately 3 mm samples of the infected blood spotted cards were removed using a sterile punch and placed in a microcentrifuge tube. Samples were rinsed in 500 μl of nuclease free water by vortexing three times for 5 s. Samples were centrifuged briefly and water removed by pipetting; 50 μl of sterile water was added, and samples were heated at 95°C for 30 min followed by brief vortexing. Tubes were centrifuged briefly to collect the sample for DNA detection.

### 2.6 Extraction and testing of ASFV DNA from multiple isolates grown in cell culture

#### 2.6.1 Archived ASFV DNA samples

DNA from known ASFV isolates Tengani/60 (genotype V), Malawi Lil‐20/1, Rhodesia (VIII), E75 and South Africa were historically stored samples. ASFV Rhodesia and ASFV South Africa isolates were originally obtained from a warthog (1954) and field ticks (1996), respectively.

#### 2.6.2 DNA extraction from BA71V cell culture grown virus

ASFV BA71V strain was previously adapted to grow in Vero cell cultures (Yanez et al., 1995). Viral DNA was extracted from infected cell culture using a modified Hirt method.

### 2.7 Real‐time PCR

Real‐time PCR was performed on an Applied Biosystems 7500 (Thermo Fisher) using primers and protocol as previously published (Zsak et al., 2005).

### 2.8 Detection of ASFV DNA using C‐SAND™ Assay kits

ASFV C‐SAND™ Assay kits (MatMaCorp), Standard and High Sensitivity, comprise three probe sets, ASF1, ASF2 and ASF3, that target conserved regions of ASFV genes 9GL, p72 and K205R, respectively. A fourth probe set, an internal positive control to detect *Sus scrofra* cytochrome b, is also included. All reagents are provided in the assay kits, consisting of lyophilized master mixes that are reconstituted and added to the reaction tubes. Amplification and detection steps are performed on the Solas 8 device.

#### 2.8.1 Detection of ASFV DNA using standard kit

Detection using the Standard ASFV C‐SAND™ Assay kit was performed in accordance with manufacturer's instructions. Results are called automatically, in real‐time, and a PDF report with final results is generated at the end of the run.

#### 2.8.2 Detection of ASFV DNA using High‐Sensitivity ASFV C‐SAND™ assay kit

Detection using the High‐Sensitivity ASFV C‐SAND™ Assay kit was performed in accordance with manufacturer's instructions. The pre‐amplification step for the High‐Sensitivity assay can now be performed on the Solas 8 without the need for a separate thermocycler. For the additional amplification step, 20 μl of Buffer S1 was added to each tube containing a lyophilized reagent pellet. After the pellets dissolved completely, 2 μl of DNA sample was added. Tubes were briefly mixed by tapping, and reagents were pooled at the bottom of the tube by tapping on the counter. For this study, tubes were placed in a GeneAmp PCR systems 9700 thermocycler (Applied Biosystems) with the following protocol: denaturation at 95°C for 3 min followed by 30 cycles of denaturation at 95°C for 10 s and annealing/amplification at 58°C for 15 s. After completion of the amplifications, an S2 reaction pellet was added to each tube before placing the tube strip in the Solas 8®.

## 3 RESULTS

### 3.1 Extraction of ASFV DNA using MagicTip™ kits

Using MagicTip™ DNA isolation kits, we isolated ASFV DNA from 99% (214/217) of samples, including blood, oral fluid and eight tissue types as detected by real‐time PCR. An additional nine samples were extracted from blood cards using manufactured suggested methodologies resulting in a total of 226 samples for testing. Tested tissues exhibited a range of Ct scores when evaluated by traditional real‐time PCR (Table 1). DNA extracted from spleen tissue had the lowest average Ct, 22.4 ± 3.1, indicative of high viral abundance. All samples except oral fluid had Ct scores overlapping those of spleen samples, suggesting that while spleen is an ideal tissue for ASFV detection, other tissue sources can be used when spleen samples are not available. Eight of 12 sample types demonstrated a difference of 10 or more between minimum and maximum obtained Ct values (Table 1), the likely result of individual swine variability as some individuals returned consistently higher (ET#38) or lower (ET#41) Ct scores across sample types (Table 2).

**TABLE 1:** Real‐time PCR of ASFV DNA extracted using the MagicTip kit

Sample	*n* (individuals)	*n* (samples)	Average Ct ± SD	Range
Blood	5	14	28.3 ± 3.4	23.6–36.1
Blood card	8	9	26.3 ± 4.8	21.3–37.5
Bone marrow	10	32	27.7 ± 3.0	21.0–35.2
Liver	5	5	26.0 ± 2.6	23.1–29.5
Lymph node	5	27	25.9 ± 4.6	17.5–35.5
Muscle	10	34	28.8 ± 3.5	22.5–36.4
Oral fluid (chew rope)	N/A^†^	4	36.3 ± 4.7^‡^	33.0 to > 41.0
Oral fluid (oral swab)	9	9	31.5 ± 3.2	26.8–36.2
Pinna (ear)	6	23	29.7 ± 4.1^‡^	24.4 to > 41.0
Skin	5	5	28.5 ± 2.5	25.7–32.4
Spleen	11	36	22.4 ± 3.1	18.4–32.6
Tonsil	5	27	26.5 ± 3.9	20.1–35.6

^†^Chew ropes were placed in the pen with ASFV infected animals and represent a pooled sample of those individuals that chewed the rope.

^‡^Samples not detected by RT‐PCR excluded from average Ct.

**TABLE 2:** Comparison of average Ct scores in tissue samples for individual swine infected with ASFV

Tissue	Individuals											Median	Range
	ET#38	ET#39	ET#40	ET#41	ET#42	ET#41^A^	ET#92	ET#93	ET#94	ET#95	ET#96		
Blood	35.0 (2)	27.7 (3)	28.8 (3)	25.0 (3)	26.9 (3)	N/A	N/A	N/A	N/A	N/A	N/A	27.7	25.0–35.0
Bone marrow	30.6 (1)	29.0 (1)	30.3 (1)	27.1 (1)	27.3 (2)	N/A	27.3 (6)	29.0 (7)	27.4 (4)	28.3 (4)	24.9 (5)	27.8	24.9– 30.6
Liver	29.5 (1)	27.4 (1)	25.9 (1)	23.1 (1)	24.0 (1)	N/A	N/A	N/A	N/A	N/A	N/A	25.9	23.1–29.5
Lymph node	N/A	N/A	N/A	N/A	N/A	N/A	25.7 (8)	27.5 (7)	25.1 (4)	24.6 (4)	25.9 (4)	25.7	24.6–27.5
Muscle	35.2 (1)	31.7 (1)	32.6 (1)	N/A	29.0 (1)	29.3 (1)	27.7 (9)	29.1 (7)	29.7 (4)	26.2 (4)	28.8 (5)	29.2	26.2–35.2
Oral swab	N/A	N/A	N/A	N/A	N/A	N/A	28.3 (1)	31.4 (2)	36.2 (1)	30.1 (1)	31.8 (1)	31.4	28.3–36.2
Pinna (ear)	34.7 (4)^†^	29.4 (5)	33.7 (4)	26.4 (4)	27.0 (5)	26.7 (1)	N/A	N/A	N/A	N/A	N/A	28.2	26.4–34.7
Skin	32.4 (1)	28.5 (1)	28.7 (1)	25.8 (1)	27.3 (1)	N/A	N/A	N/A	N/A	N/A	N/A	28.5	25.8–32.4
Spleen	30.6 (2)	24.7 (2)	25.0 (2)	24.8 (1)	21.0 (1)	21.1 (1)	21.4 (8)	21.6 (7)	22.0 (4)	20.0 (4)	22.1 (4)	22.0	20.0–30.6
Tonsil	N/A	N/A	N/A	N/A	N/A	N/A	26.2 (8)	27.5 (7)	28.5 (4)	26.7 (4)	23.3 (4)	26.7	23.3–28.5

*Note*: Each individual DNA extraction was analysed in triplicate by real‐time PCR. The number of replicate DNA extractions from single tissue source are in parenthesis. N/A indicates that sample was not obtained from that individual.

^†^One of four tested samples was not detected by RT‐PCR.

Viral DNA extracted from oral fluid returned the highest Ct scores, indicative of low viral abundance (Table 1), and included two samples without detectable viral DNA (data not shown). To ensure these results were not extraction methodology dependent, both MagicTip™ and MagMax™ extraction methodologies were performed on a single oral fluid sample. For this sample, DNA extracted by the MagicTip™ oral fluid kit had a Ct of 31.0 ± 0.04 while MagMax™ extracted DNA had a Ct of 31.5 ± 0.2 (data not shown), demonstrating the difficulty of ASFV detection in oral fluid is independent of these two extraction methodologies.

### 3.2 Detection of ASFV DNA using C‐SAND™ assay

**FIGURE 1:**
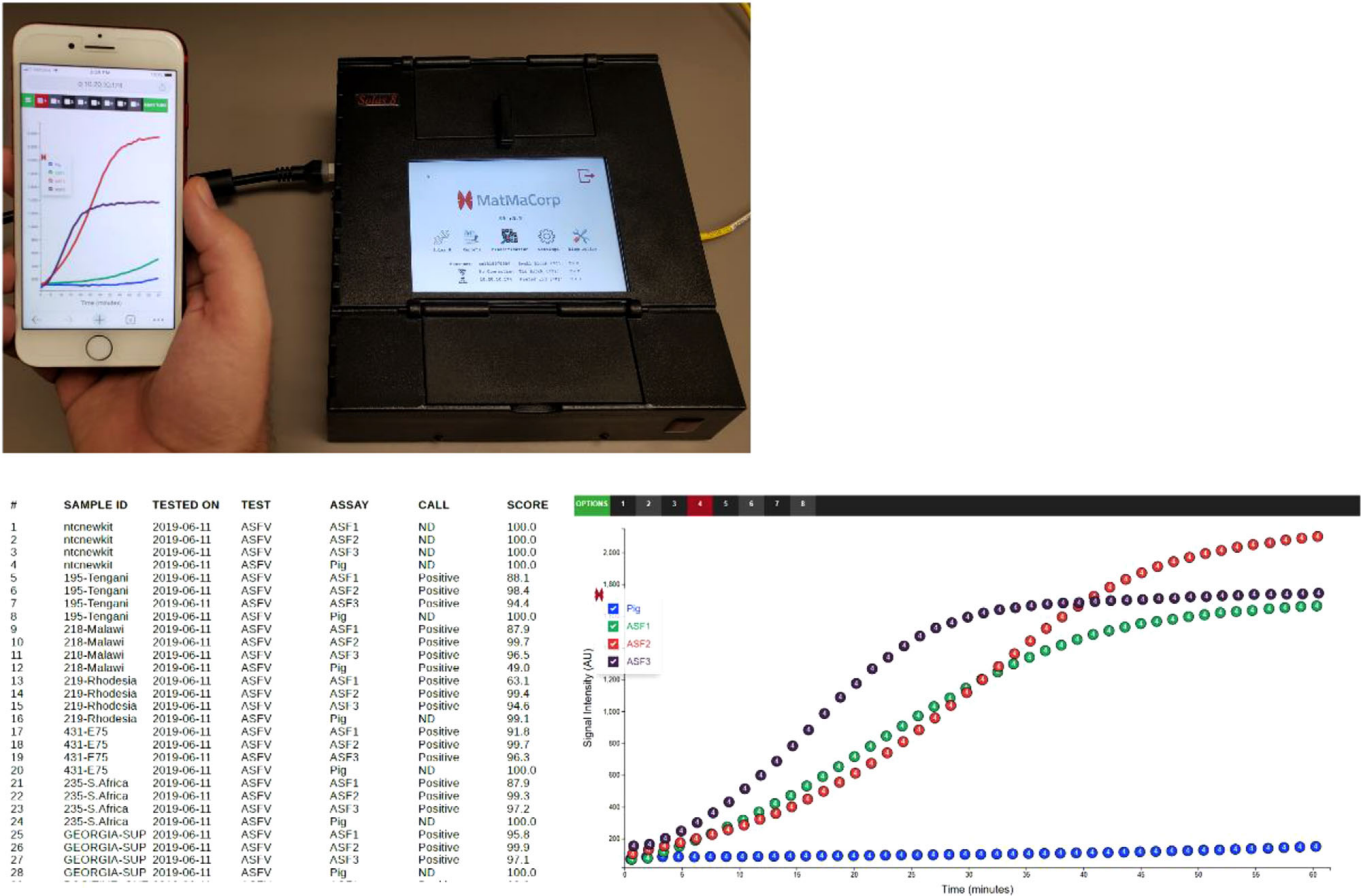
The Solas 8 contains a built‐in router allowing connections with smart phones and similar mobile devices. Results from C‐SAND™ assays can be visualized on the Solas 8 directly as text while connection to a smart phone allows visualization of probe amplification kinetics and comparison of signal intensities among samples

Two ASFV C‐SAND™ Assay kits, Standard and High Sensitivity, were evaluated with DNA extracted from multiple tissues. Kits utilized identical probe sets but differ in the inclusion of a pre‐amplification step to improve assay sensitivity. No false‐positive signals were obtained with either Standard or High‐Sensitivity assays when ASFV‐negative tissues were analysed (Supplemental Table S1). Results from the C‐SAND™ Assays can be presented on the Solas 8 directly as text, or by connecting a smart phone device to the built in router allowing visualization of amplification kinetics for each probe in addition to textual results (Figure 1). Real‐time PCR and individual probe results for all ASF positive samples can be found in Supplemental Table S2.

#### 3.2.1 Detection using standard ASFV C‐SAND™ assay

We tested 123 samples using the Standard assay (Table 3). Blood was an ideal sample for ASFV detection with the Standard assay because it is readily obtained from live animals and gave a 93% (13/14 samples) ASFV detection rate. ASFV detection rates from spleen, liver, pinna and bone marrow tissues were 85% (11/13), 80% (4/5), 74% (17/23) and 64% (14/22), respectively (Table 3).

**TABLE 3:** Performance of Standard and High‐Sensitivity ASFV C‐SAND™ assays in relation to tissue type and average Ct score obtained by real‐time PCR

		Standard			High Sensitivity		
Sample	Call	*N*	Avg. Ct ± SD	Range	*N*	Avg. Ct ± SD	Range
Blood	Positive	13	27.7 ± 2.6	23.6–33.9	13	27.7 ± 2.6	23.6–33.9
	Not detected	1	36.1	–	1	36.1	–
Bone marrow	Positive	14	26.3 ± 1.9	22.7–30.3	15	28.9 ± 3.0	23.8–35.2
	Not detected	8	29.0 ± 1.6	26.9–31.4	1	26.9	–
Liver	Positive	4	25.1 ± 1.9	23.1–27.4	5	26.0 ± 2.6	23.1–29.5
	Not detected	1	29.5	–	0	–	–
Lymph node	Positive	1	20.8	–	4	22.6 ± 1.6	20.8–24.0
	Not detected	4	23.6 ± 4.8	17.5–29.2	3	24.8 ± 6.4	17.5–29.2
Muscle	Positive	13	26.5 ± 2.0	22.5–29.9	24	29.2 ± 3.7	22.5–36.4
	Not detected	14	29.5 ± 3.6	23.6–36.4	1	27.3	–
Oral fluid (swab)	Positive	4	29.2 ± 2.1	26.8–31.7	4	33.5 ± 3.1	30.1–36.2
	Not detected	3	34.9 ± 2.0	32.7–36.2	0	–	–
Pinna (ear)	Positive	17	28.2 ± 2.5	24.4–31.6	16	28.5 ± 2.5	24.4–31.6
	Not detected	6	34.8 ± 4.9	29.3 to > 41.0	5	33.7 ± 6.4	25.2–41.0
Skin	Positive	2	30.6	28.7–32.4	3	29.5 ± 2.6	27.3–32.4
	Not detected	2	27.1	25.7–28.5	2	27.1	25.7–28.5
Spleen	Positive	11	23.4 ± 2.8	18.7–28.5	13	22.5 ± 3.2	18.4–28.5
	Not detected	2	25.5	18.4–32.6	1	32.6	–
Tonsil	Positive	0	–	–	5	23.4 ± 2.7	20.1–27.5
	Not detected	3	26.4 ± 2.4	23.6–28.0	2	27.9	27.7–28.0
Blood card	Positive	0	–	–	9	27.7 ± 2.6	21.3–37.5
	Not detected	0	–	–	0	–	–
Oral fluid (rope)	Positive	0	–	–	1	33.0	–
	Not detected	0	–	–	3^†^	39.6	39.6 to > 41.0

*Notes*: Only samples with three or more Ct scores have standard deviations reported.

^†^ Two of three samples were not detectible by real‐time‐PCR.

ASFV‐positive samples undetected by the Standard assay had a higher average Ct value compared to ASFV samples of the same type that were detected (Table 3), although a definitive cut‐off was not established in this study. Some tissue types, such as tonsil and lymph node, gave poor detectability despite low Ct scores by real‐time PCR (Table 3), suggesting that tissue type may influence sensitivity.

#### 3.2.2 Detection using High‐Sensitivity ASFV C‐SAND™ assay

We tested 132 samples using the High‐Sensitivity assay, including nine with DNA extracted using the blood card methodology. The High‐Sensitivity assay was more sensitive than the Standard assay, broadening the number of samples giving consistent detection. ASFV DNA was detected in over 90% of the blood, bone marrow, liver, muscle, oral swabs and spleen samples (Table 3). The High‐Sensitivity assay also enhanced detection in tonsil and lymph node tissues, poorly detected with the Standard assay. Blood card samples are a convenient method for field collection, transport and sample retention by diagnostic laboratories that can provide confirmatory testing using validated RT‐PCR procedures. When DNA was extracted from blood cards, the High‐Sensitivity assay detected ASFV DNA in all nine samples tested.

A subset of 100 samples was assayed with both the Standard and High‐Sensitivity assays for direct comparisons (Table 4). The High‐Sensitivity assay enhanced ASFV DNA detectability in all samples except blood (93%), pinna (76%) and skin (50%). The improvement in sensitivity using the High‐Sensitivity assay was most notable for oral swabs and tonsil samples where only samples where ASFV was undetected using the Standard assay were tested. The High‐Sensitivity assay also demonstrated a significant improvement for ASFV detection in muscle tissue, from 38% to 95%, likely of practical importance to pork processing facilities and food safety testing.

**TABLE 4:** Comparison of Standard and High‐Sensitivity ASFV C‐SAND™ Assays sensitivity on same sourced samples

Sample	*n* (individuals)	*n* (samples)	Standard	High Sensitivity
Blood	5	14	93%	93%
Bone marrow	9	14	50%	93%
Liver	5	5	80%	100%
Lymph node	3	5	20%	60%
Muscle	10	21	38%	95%
Oral fluid (swab)	2	2	0%	100%
Pinna (ear)	6	21	76%	76%
Skin	4	4	50%	50%
Spleen	7	11	82%	91%
Tonsil	2	3	0%	67%

### 3.3 Testing of samples in advanced stage decomposition

Determination of a likely cause of death in ASF suspect field cases is critical for outbreak response and containment. Unlike samples obtained under controlled laboratory conditions, field samples may be in various states of decay or missing tissues due to scavengers, limiting quality sample availability. To simulate relative high temperature field conditions, muscle and bone marrow samples were stored at 37°C in the laboratory. Within 24 h post‐incubation, samples demonstrated putrid qualities associated with an advanced state of decay.

Average Ct scores for extracted DNA increased over time for both tissue types (Table 5), but remained detectable by real‐time PCR in all samples. Both the Standard and High‐Sensitivity assays detected ASFV in muscle tissue after three days of incubation. Following bone marrow sample incubation for 24–48 h, the Standard assay failed to detect ASFV DNA while the High‐Sensitivity assay results remained positive.

**TABLE 5:** Effect of decomposition on ASFV DNA detection

Tissue	Days post‐harvest	Average Ct ± SD	Standard	High Sensitivity
Muscle	0	25.0 ± 0.1	Positive	Positive
Muscle	1	26.7 ± 0.1	Positive	Not tested
Muscle	3	28.9 ± 0.3	Positive	Positive
Bone marrow	0	26.2 ± 0.6	Positive	Positive
Bone marrow	1	27.7 ± 0.1	Not detected	Positive
Bone marrow	2	30.3 ± 0.1	Not detected	Positive

### 3.4 Versatility of C‐SAND™ Assay kit probes with additional ASFV strains

While ASFV strain Georgia represents the current epizootic p72 genotype II strain, numerous other ASFV circulating strains exist, particularly in sub‐Saharan Africa. We tested the robustness of the C‐SAND™ Assay probes against six different strains of ASFV, field isolated strains, Tengeni (V), Malawi, Rhodesia and South Africa (XXI), as well as cell culture adapted strains, E75 and BA71V. All three probes identified all tested strains, demonstrating that the assay has broad strain applicability, data not shown.

## 4 DISCUSSION

This report demonstrated the capability of the MatMaCorp ASFV detection system, utilizing associated MagicTip™ and C‐SAND™ kits, to extract and detect ASFV DNA from a variety of swine sample types. This includes the OIE recognized traditional ASFV diagnostic samples, blood and spleen, as well as non‐traditional samples, such as pinna, liver, muscle and bone marrow.

Using MagicTip™ kits, we extracted ASFV DNA from 214/217 samples, and an additional 9/9 samples were detected using a blood card extraction methodology. Two of the three failures were from oral fluid collected by chew rope, which also proved difficult to extract by other methods, possibly due to the low ASFV abundance. The third extraction failure was a pinna sample from an animal, ET# 38, which showed higher Ct counts in all tested samples relative to the other pigs and samples. Additional extractions from the same pinna sample produced detectable DNA, suggesting that conducting more than one extraction from a sample may reduce the occurrence of detection failures.

Two C‐SAND™ kits, Standard and High Sensitivity, were tested for their ability to detect ASFV DNA. The Standard assay was proficient at detecting ASFV DNA in blood, spleen and liver samples. Incorporation of an additional pre‐amplification step in the High‐Sensitivity assay broadened the swine sample types with detectable ASFV DNA. ASFV in blood, bone marrow, liver, muscle and spleen was detected at a rate of 90% or higher with the High‐Sensitivity assay. Both assays rapidly detected ASFV: the Standard assay within 2 h and the High‐Sensitivity assay by 3 h. For both Standard and High‐Sensitivity assays, real‐time monitoring detected positive samples prior to completion of the full amplification time, shortening the time needed for detection in most cases. The broad range of sample types, including bone marrow and muscle, coupled with the rapid assay time, has the potential to be a relatively quick and effective way to test food grade samples at pork processing facilities or carcass samples during outbreak investigations.

The versatility of the MatMaCorp ASFV detection system for a variety of samples suggests its utility for field investigations. During outbreak investigations, there is a need to test both suspect cases and suspicious deaths in the field. Blood and pinna samples represent samples that can be taken relatively easily from live pigs with little or no detriment to their long‐term health.

Among samples collected at necropsy, spleen had the lowest average Ct score by real‐time PCR and a high rate of detection with both the Standard and High‐Sensitivity assays, highlighting it as the preferred tissue for ASFV detection. Although the use of spleen samples for the detection of ASFV is preferred, such samples may be unavailable or not easily accessible in a field situation, particularly when dealing with the carcass of a wild boar or feral hog that might be of significant size and in the process of decomposition. We demonstrated that this assay was effective with both muscle and bone marrow samples incubated at elevated temperatures to replicate several days of decomposition.

The MatMaCorp ASFV detection system offers the potential for pre‐screening or triage of field samples, reducing the burden on ASF diagnostic laboratories during outbreak response investigations, especially in situations where transport of samples to centralized diagnostic laboratories is impractical. In particular, the ability of the portable MatMaCorp ASFV detection system to be utilized on carcasses during early decomposition would be useful in a wild suid ASF outbreak investigation.

Rapid and effective diagnosis of ASF is critical to containment of any outbreak. All three probes utilized in the C‐SAND™ kits were reactive to six additional ASFV p72 strains of ASFV tested, indicating that other strains may be detectable by one or more of the current probe sets. This is critical for ASFV active and passive surveillance strategies, particularly as new ASFV strains emerge. While the presence of three detection channels allows for the potential of multiplexing with other pathogens utilizing all three probes to target ASFV better prevents false‐negative results on a single sample.

Utilizing both the Standard and High‐Sensitivity C‐SAND™ assays, combined with sample‐specific MagicTip DNA Isolation kits, the Solas 8® was highly effective in speed and sensitivity for ASFV detection. We expect that the rapid detection time of ASFV from various sample types will be useful for screening in food processing facilities, customs checkpoints or in remote field locations, all of which may present limited sample types for testing.

## CONFLICT OF INTEREST

Authors M. Carrie, H. Piscatelli, A. Hangman and D. Petrik are employees of MatMaCorp. The remaining authors declare they have no competing interests.

## ETHICS STATEMENT

The ethical policies of the journal, as noted in the journal guidelines, were adhered to. All samples were obtained from swine studies that were conducted after approval by the Plum Island Animal Disease Center Institutional Animal Care and Use Committee.

## Supporting information

Supporting Information

Supporting Information

## Data Availability

The data that support the findings of this study are available in the supplementary material of this article.
